# Hypoxic pulmonary vasoconstriction does not limit maximal exercise capacity in healthy volunteers breathing 12% oxygen at sea level

**DOI:** 10.14814/phy2.15944

**Published:** 2024-02-16

**Authors:** Nick P. Talbot, Hung‐Yuan Cheng, Helen Hanstock, Thomas G. Smith, Keith L. Dorrington, Peter A. Robbins

**Affiliations:** ^1^ Department of Physiology, Anatomy and Genetics University of Oxford Oxford UK; ^2^ Translational Health Sciences, Bristol Medical School University of Bristol Bristol UK; ^3^ Swedish Winter Sports Research Centre, Department of Health Sciences Mid Sweden University Östersund Sweden; ^4^ Centre for Human and Applied Physiological Sciences King's College London London UK; ^5^ Guy's and St Thomas' NHS Foundation Trust London UK

**Keywords:** Exercise, hypoxia, hypoxic pulmonary vasoconstriction, iron

## Abstract

Maximal exercise capacity is reduced at altitude or during hypoxia at sea level. It has been suggested that this might reflect increased right ventricular afterload due to hypoxic pulmonary vasoconstriction. We have shown previously that the pulmonary vascular sensitivity to hypoxia is enhanced by sustained isocapnic hypoxia, and inhibited by intravenous iron. In this study, we tested the hypothesis that elevated pulmonary artery pressure contributes to exercise limitation during acute hypoxia. Twelve healthy volunteers performed incremental exercise tests to exhaustion breathing 12% oxygen, before and after sustained (8‐h) isocapnic hypoxia at sea level. Intravenous iron sucrose (*n* = 6) or saline placebo (*n* = 6) was administered immediately before the sustained hypoxia. In the placebo group, there was a substantial (12.6 ± 1.5 mmHg) rise in systolic pulmonary artery pressure (SPAP) during sustained hypoxia, but no associated fall in maximal exercise capacity breathing 12% oxygen. In the iron group, the rise in SPAP during sustained hypoxia was markedly reduced (3.4 ± 1.0 mmHg). There was a small rise in maximal exercise capacity following sustained hypoxia within the iron group, but no overall effect of iron, compared with saline. These results do not support the hypothesis that elevated SPAP inhibits maximal exercise capacity during acute hypoxia in healthy volunteers.

## 1 INTRODUCTION

Maximal aerobic exercise capacity (*V* o_2_max) is decreased in healthy individuals at high altitude or during hypoxia at sea level (Fulco et al., 1998; Pugh et al., 1964), and this is associated with a reduction in maximal cardiac output (Cymerman et al., 1989; Pugh et al., 1964; Reeves et al., 1990). However, the extent to which these observations are causally related remains uncertain (Anholm & Foster, 2011; Wagner, 2010).

It has been suggested that hypoxic pulmonary vasoconstriction might limit *V* o_2_max at high altitude by increasing pulmonary vascular resistance and therefore right ventricular afterload (Naeije, 2011; Naeije & Chesler, 2012). In support of this possibility, aerobic exercise capacity both at sea level and at altitude appears to correlate with pulmonary vascular distensibility (Lalande et al., 2012; Pavelescu et al., 2013) and pulmonary vasodilators have been shown in some studies to improve *V*O_2_max during acute hypoxia or at high altitude (Faoro et al., 2009; Ghofrani et al., 2004; Naeije et al., 2010). In opposition, supplemental oxygen is reported by some authors to return *V*O_2_max to sea level values during prolonged exposure to hypoxia without influencing pulmonary vascular resistance (Groves et al., 1987; Pugh et al., 1964), and modeling studies suggest that *V*O_2_max is largely independent of maximal cardiac output during hypoxia, as any rise in convective oxygen delivery is offset by diffusive limitation across capillaries in the lungs and muscle (Wagner, 1996, 2010).

We have previously demonstrated that hypoxic pulmonary vasoconstriction in healthy humans exposed to sustained eucapnic hypoxia consists of an initial modest rise in pulmonary artery pressure that begins within seconds, followed by a more gradual rise in pulmonary artery pressure that develops over 4–8 h (Dorrington et al., 1997; Talbot et al., 2005). Upon return to normoxia, the pulmonary pressure initially falls rapidly, but remains elevated above baseline for at least 2 h, and during this period re‐exposure to acute hypoxia elicits an exaggerated acute rise in pulmonary artery pressure, suggesting sensitization of the pulmonary vascular smooth muscle cells to hypoxia (Dorrington et al., 1997; Frise & Robbins, 2015). The mechanism of this sensitization remains uncertain, but it is likely to be under the control the hypoxia‐inducible factor (HIF) transcriptional pathway, which is known to be regulated not only by oxygen, but also by cellular iron availability (Knowles et al., 2003; Wang & Semenza, 1993). Accordingly, intravenous iron infusion has been shown to inhibit the pulmonary vascular response to hypoxia both at sea level and at altitude (Patrician et al., 2022; Smith et al., 2008, 2009; Willie et al., 2021).

In the current study, we hypothesized that the elevation of pulmonary artery pressure during sustained hypoxia would reduce maximal exercise capacity during hypoxia, and that this effect would be mitigated by prior iron infusion. To explore this hypothesis, we measured *V* o_2_max in healthy volunteers breathing 12% oxygen, before and after exposure to sustained (8 h) isocapnic hypoxia, with or without prior infusion of intravenous iron.

## 2 METHODS

### 2.1 Participants

Twelve volunteers participated in the study (8 male, 4 female, age 25 ± 5 years). All were healthy non‐smokers with no history of respiratory or cardiovascular disease. Baseline characteristics are provided in Table 1. All participants provide written, informed consent. The study was approved by the National Research Ethics Service (reference 10/H0604/73), United Kingdom, and performed in accordance with principles of the Declaration of Helsinki.

**TABLE 1:** Baseline characteristics. Iron indices and hemodynamic parameters were measured at the baseline visit (before any iron supplementation). VO_2_max was unavailable for two volunteers at baseline (one in each group) due to technical difficulties with mass spectrometry. For all other measurements *n* = 6 in each group and all values are shown as mean ± SD.

	Iron group	Placebo group
Age (years)	25 ± 4	25 ± 6
Weight (kg)	76 ± 16	76 ± 15
Height (cm)	179 ± 10	178 ± 14
BMI (kg/m^2^)	23 ± 3	24 ± 3
Hemoglobin (g/L)	145 ± 10	143 ± 10
Mean cell volume (fL)	90 ± 5	91 ± 5
Ferritin (μg/L)	112 ± 83	65 ± 54
Serum iron (μmol/L)	21 ± 6	22 ± 11
Transferrin (g/L)	2.7 ± 0.5	2.7 ± 0.3
Transferrin saturation (%)	37 ± 15	37 ± 18
Arterial oxyhemoglobin saturation (%)	98 ± 1	98 ± 1
Mean arterial blood pressure (mmHg)	91 ± 5	86 ± 8
Systolic pulmonary artery pressure (mmHg)	24.3 ± 4.5	24.2 ± 2.4
Cardiac output (L/min)	5.0 ± 1.1	5.3 ± 0.5
*V*O_2_max (mL/min/kg)	47.9 ± 8.2	46.0 ± 8.5
Peak work rate (W)	301 ± 71	299 ± 81

Abbreviations: BMI, body mass index; *V*O_2_max, maximal oxygen uptake, measured breathing air.

### 2.2 Protocol

**FIGURE 1:**
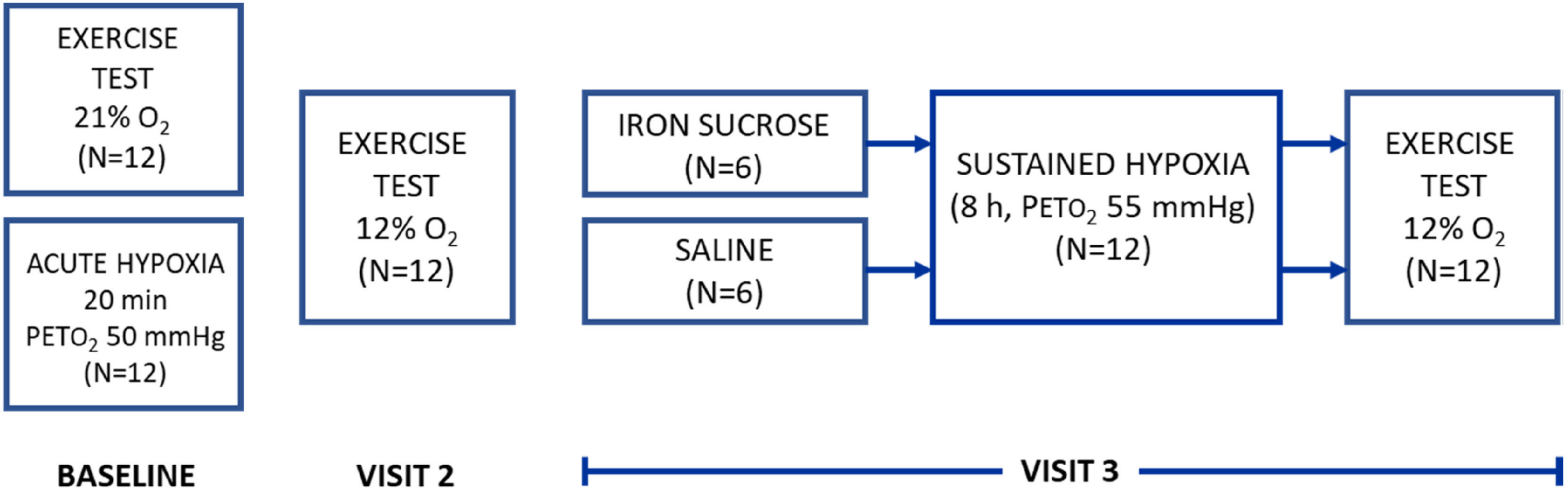
Study design. The baseline visit and visit 2 were identical for all 12 participants. On the morning of visit 3, participants were randomized to receive an infusion of either iron sucrose (*n* = 6) or saline (*n* = 6), shortly before being exposed to 8 h of sustained hypoxia followed by an exercise test breathing 12% oxygen. PETO_2_, end‐tidal partial pressure of oxygen.

The experimental protocol included three visits to the laboratory for each participant, and is summarized in Figure 1. The *baseline* visit included measurements of iron status (serum iron, transferrin, ferritin and hemoglobin concentrations, and mean cell volume), measurement of the pulmonary vascular response during acute (20 min) isocapnic hypoxia, and an incremental exercise test to exhaustion breathing room air in the laboratory (altitude 60 m). *Visit 2* took place at least a week later in the late afternoon/evening, and consisted of an incremental exercise test to exhaustion while breathing 12% oxygen, equivalent to an altitude of ~4700 m. *Visit 3* took place 2–3 days after visit 2. In the morning on visit 3, participants were randomized to receive an intravenous infusion of either iron sucrose (*n* = 4 males, 2 females) or saline placebo (*n* = 4 males, 2 females). Each participant was then exposed to 8 h of isocapnic hypoxia in a custom‐built normobaric chamber, during which the systolic pulmonary artery pressure (SPAP) was measured by echocardiography every 1–2 h. This was followed within 20 min by an incremental exercise test to exhaustion breathing 12% oxygen, identical to the test undertaken during visit 2.

### 2.3 Exposure to acute hypoxia at rest

To quantify the pulmonary vascular response to acute hypoxia at baseline and confirm echocardiographic windows for measurement of pulmonary artery pressure during hypoxia at subsequent visits, participants were exposed during the baseline visit to a short period of isocapnic hypoxia while reclining in the left lateral position and breathing through a mouthpiece. Gas control was achieved by means of dynamic end‐tidal forcing (Robbins et al., 1982b), and the exposure consisted of an initial 5‐min period of normoxia (end‐tidal partial pressure of oxygen, PETO_2_, 100 mmHg), followed by 20 min of hypoxia (PETO_2_ 50 mmHg), and then by a further 10 min of normoxia. The end‐tidal partial pressure of carbon dioxide (PETCO_2_) was maintained close to each volunteer's baseline value.

### 2.4 Exercise testing

Exercise tests were performed using a modified electrically‐braked cycle ergometer (Mijnhardt KEM3, Cardiokinetics Salford, UK). Participants breathed through a mouthpiece with their nose occluded. Respired gases were sampled through a fine catheter and the composition analyzed continuously by mass spectrometry (Airspec MGA 3000, UK). Respiratory volumes were measured using a combination of a turbine (Cardiokinetics Ltd, UK) and pneumotachograph (Fleisch, Switzerland), as previously reported (Robbins et al., 1982a). Ventilation is reported at body temperature and pressure saturated (BTPS). Oxygen consumption was calculated breath‐by‐breath as the difference between the inspired and expired oxygen content. Oxyhemoglobin saturation was measured continuously using a pulse oximeter and heart rate was measured with a three‐lead electrocardiogram (ECG).

**FIGURE 2:**
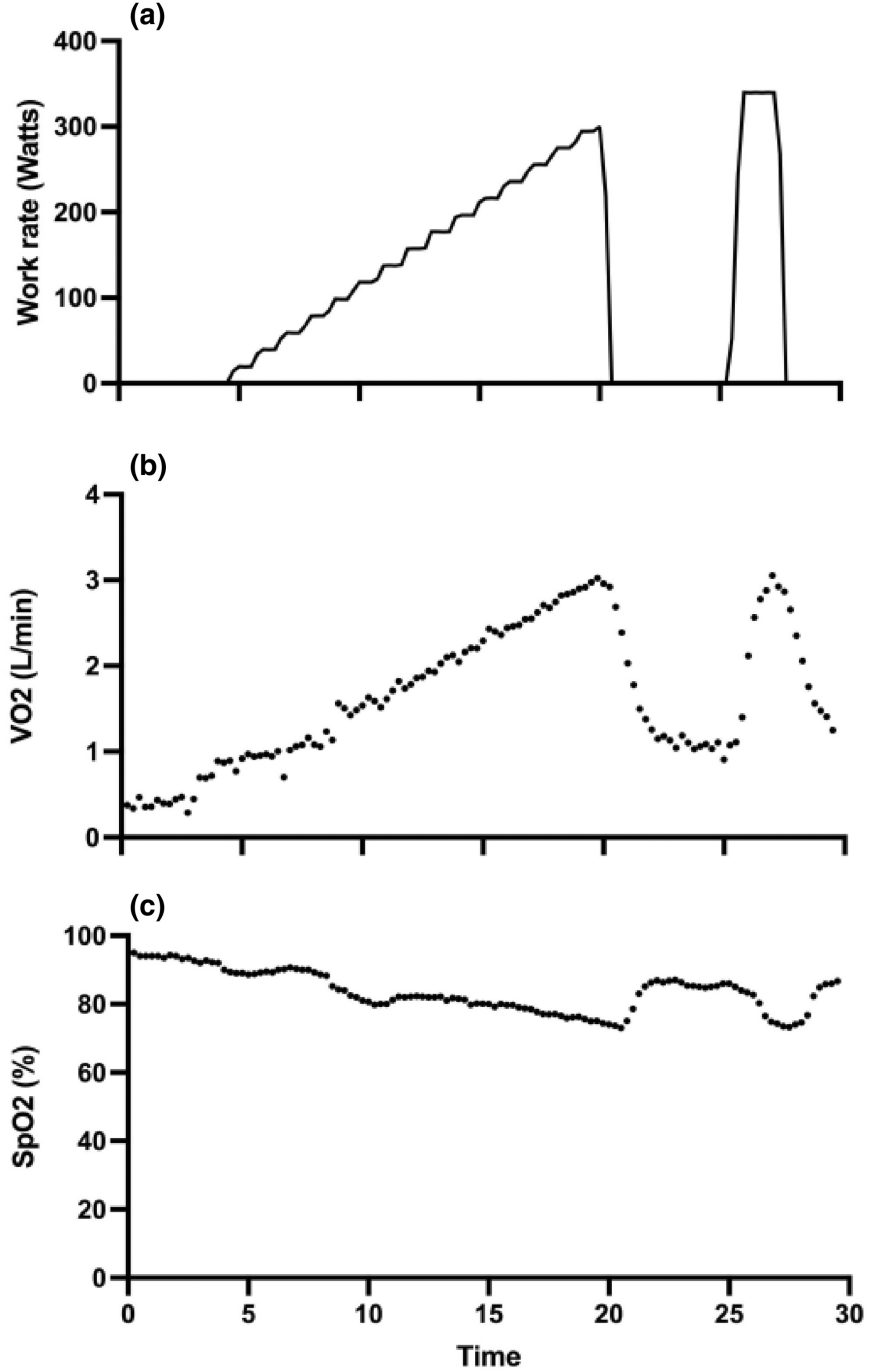
Example exercise test breathing 12% oxygen. (a) Work rate, (b) oxygen uptake, VO_2_, (c) arterial oxyhemoglobin saturation, SpO_2_. This tracing shows unloaded cycling breathing air for 3 min, before switching to inspired gas containing 12% oxygen, balance nitrogen. Unloaded cycling continued for 1 min, and thereafter work load was increased by 20 W every min until exhaustion, defined as an inability to maintain the pedaling frequency of 60 per min. At the point of exhaustion, following the protocol of Rossiter et al. (2006), the ergometer was unloaded for 5 min before the workload was returned to a value 105% of the previous maximum for a period of up to 3 min, or until the point of exhaustion.

At the baseline visit, the exercise test was undertaken breathing air. During visits 2 and 3, the exercise tests were undertaken breathing 12% oxygen. The exercise protocol during each visit was otherwise the same. After 1 min of unloaded cycling, the workload was increased by 20 W/min until exhaustion, defined as an inability to maintain the required pedaling frequency of 60 revolutions per min. At that point, using a previously‐reported protocol (Rossiter et al., 2006), the ergometer was unloaded for 5 min, before the workload was returned to a value 105% of the previous maximum for a period of up to 3 min, or until the point of exhaustion. The *V* o_2_max was defined as the mean oxygen uptake over the 10 breaths immediately prior to exhaustion, and the higher of the two peaks was used in the study analysis. Peak workload was calculated using the duration of time spent exercising at the final increment at the point of exhaustion, based on an established approach (Kuipers et al., 1985). An example exercise test performed breathing 12% oxygen during visit 2 is provided in Figure 2.

At visits 2 and 3, venous blood was sampled every minute during exercise tests, using an indwelling cannula in the antecubital fossa, for measurement of blood lactate concentration. No blood sampling was performed during exercise at the baseline visit.

In two participants at the baseline visit, *V* o_2_max could not be measured due to technical problems with mass spectrometry, but peak work load and other exercise parameters were recorded. In one participant at visit 2 and one participant at visit 3, oxyhemoglobin saturation could not be accurately measured due to poor signal. In two participants at visit 3, heart rate could not be accurately recorded due to poor signal.

### 2.5 Iron and saline infusions

On the morning of visit 3, block randomization was used to allocate participants to the iron or saline group (*n* = 6 in each case). Those in the iron groups received an intravenous infusion of Fe(III)‐hydroxide sucrose (200 mg in 100 mL 0.9% saline, administered over 30 min; Vifor Inc, St Gallen, Switzerland). Those in the saline group received an infusion of 100 mL 0.9% saline over 30 min. Infusion were administered from behind a screen and infusion lines were taped, to ensure that participants remained unaware which infusion they had received.

### 2.6 Exposures to sustained isocapnic hypoxia

Within 30 min of iron or saline infusion at visit 3, participants were exposed to 8 h of isocapnic hypoxia in a custom‐built normobaric chamber. End‐tidal gases were controlled using a computerized prediction–correction system that has been described in detail elsewhere (Howard et al., 1995), such that the PETO_2_ was maintained at 55 mmHg and the PETCO_2_ was maintained at each individual's baseline value. Volunteers were provided with light refreshment ad libitum, and were free to move around the chamber. Every 1–2 h, volunteers were asked to rest in the left lateral position for at least 5 min, prior to assessment of pulmonary artery pressure and cardiac output using Doppler echocardiography.

### 2.7 Doppler echocardiography

During exposures to acute and sustained hypoxia at rest, Doppler echocardiography (Vivid‐*i*; GE Healthcare, UK) was used to determine the maximum systolic pressure gradient across the tricuspid valve, and the SPAP was estimated using the modified Bernoulli equation and an estimated right atrial pressure of 5 mmHg (Smith et al., 2008; Yock & Popp, 1984). Stroke volume was estimated by measuring the velocity of blood flow just below the aortic valve and the diameter of the left ventricular outflow tract at the same point, and heart rate was measured using a three‐lead ECG. Cardiac output was estimated as previously described (Balanos et al., 2005).

### 2.8 Statistical analysis

The Shapiro–Wilk test was used to assess the distribution of the data, and provided no evidence of deviation from the normal distribution for any parameter. For comparisons between groups at baseline, and between exercise parameters breathing air and 12% oxygen, unpaired or paired *t*‐tests were therefore used, respectively. Assessment of changes during exposure to acute or sustained hypoxia, and of changes in exercise parameters between visits 2 and 3, were made using repeated measures ANOVA (rmANOVA). Comparisons between iron and saline groups were also made using rmANOVA, with time or visit as a within‐participant factor and group (iron or saline) as a between‐participant factor (IBM SPSS statistics). Correlations were quantified using Pearson's correlation coefficient. Statistical significance was assumed when *p* < 0.05.

## 3 RESULTS

### 3.1 Baseline iron status and responses to hypoxia and exercise at rest

Table 1 shows that the baseline iron status for the two groups was similar. The iron indices for all participants were in the normal range, with the exception of the baseline ferritin concentration for one female participant in the iron group, who was noted incidentally to have a ferritin below the lower limit of normal, with a normal transferrin saturation and normal hemoglobin concentration. The iron group overall had a slightly higher ferritin concentration than the placebo group at baseline, but this difference was not statistically significant (*p* = 0.24).

Table 1 also includes the baseline measurements of SPAP and cardiac output, as well as *V*O_2_max and maximal work rate breathing air. Across all 12 participants, there were significant correlations between baseline hemoglobin concentration and both *V*O_2_max (*R* = 0.866, *p* < 0.001) and maximal work rate (*R* = 0.737, *p* < 0.01). Similarly, there was a significant association between ferritin concentration and baseline *V*O_2_max (*R* = 0.675, *p* < 0.05) and work rate (*R* = 0.616, *p* < 0.05).

**FIGURE 3:**
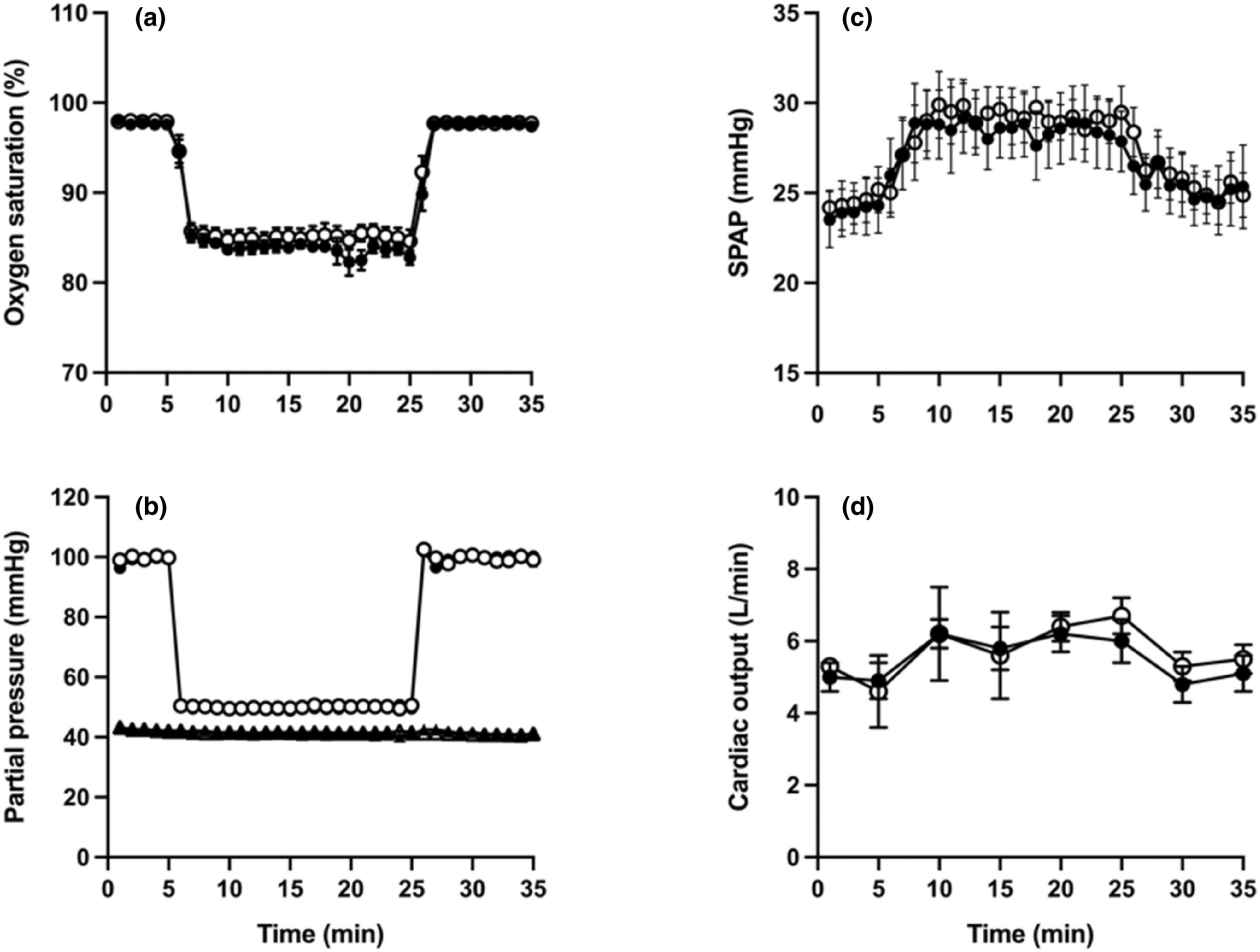
Exposure to 20 min of acute isocapnic hypoxia end‐tidal partial pressure of oxygen (PETO_2_) 50 mmHg, end tidal partial pressure of carbon dioxide (PETCO_2_) 2 mmHg above each participant's normal value. (a) Peripheral oxyhemoglobin saturation. (b) PETO_2_ (circles) and PETCO_2_ (triangles). (c, d) Systolic pulmonary artery pressure (SPAP) and cardiac output, respectively, measured by echocardiography. Symbols represent mean ± SEM. Participants subsequently in the iron group are shown with filled symbols, and those subsequently in the placebo group are shown with open symbols.

The pulmonary vascular response to acute isocapnic hypoxia is shown in Figure 3, and consists of a rapid rise in SPAP that starts within seconds of exposure to hypoxia, followed by a plateau that lasts for the duration of the 20 min exposure. The cardiac output rose with a similar time course, and changes in both SPAP and cardiac were very similar in the iron and saline groups. There was no correlation between the magnitude of the rise in SPAP during acute hypoxia and the exercise capacity breathing air at the baseline visit (for *V*O_2_max *R* = 0.154, *p* = 0.671; for peak work rate *R* = 0.02, *p* = 0.950).

### 3.2 Exercise parameters breathing 12% oxygen

Across all participants, there was a 19.4 ± 1.5% fall in maximal work rate when breathing 12% oxygen, compared with breathing air (*n* = 12, *p* < 0.001). In two participants (one in each group), *V*O_2_max could not be measured during exercise breathing air due to technical problems at the baseline visit, as above. For the remaining participants, there was a 24.1 ± 3.0% fall in *V*O_2_max (*n* = 10, *p* < 0.001) breathing 12% oxygen during visit 2, compared with air, which was accompanied by a fall in peak ventilation (118 ± 9 compared with 108 ± 10 L/min; *n* = 10, *p* < 0.01) and heart rate (170 ± 4 compared with 159 ± 4 beats/min; *n* = 10, *p* < 0.05). At *V*O_2_max, the peripheral oxyhemoglobin saturation was 95.2 ± 0.7% breathing air, and 76.8 ± 0.9% breathing 12% oxygen (*n* = 10, *p* < 0.001).

There was no significant correlation between the rise in SPAP during acute hypoxia at rest at baseline and either *V*O_2_max or maximal work rate breathing 12% oxygen at visit 2. There was also no correlation between baseline hemoglobin or ferritin concentration and *V*O_2_max breathing 12% oxygen, but both variables correlated with maximal work rate breathing 12% oxygen (*R* = 0.777, *p* < 0.01 for hemoglobin; *R* = 0.616, *p* < 0.05 for ferritin).

### 3.3 Iron infusion and pulmonary vascular response to sustained hypoxia

**FIGURE 4:**
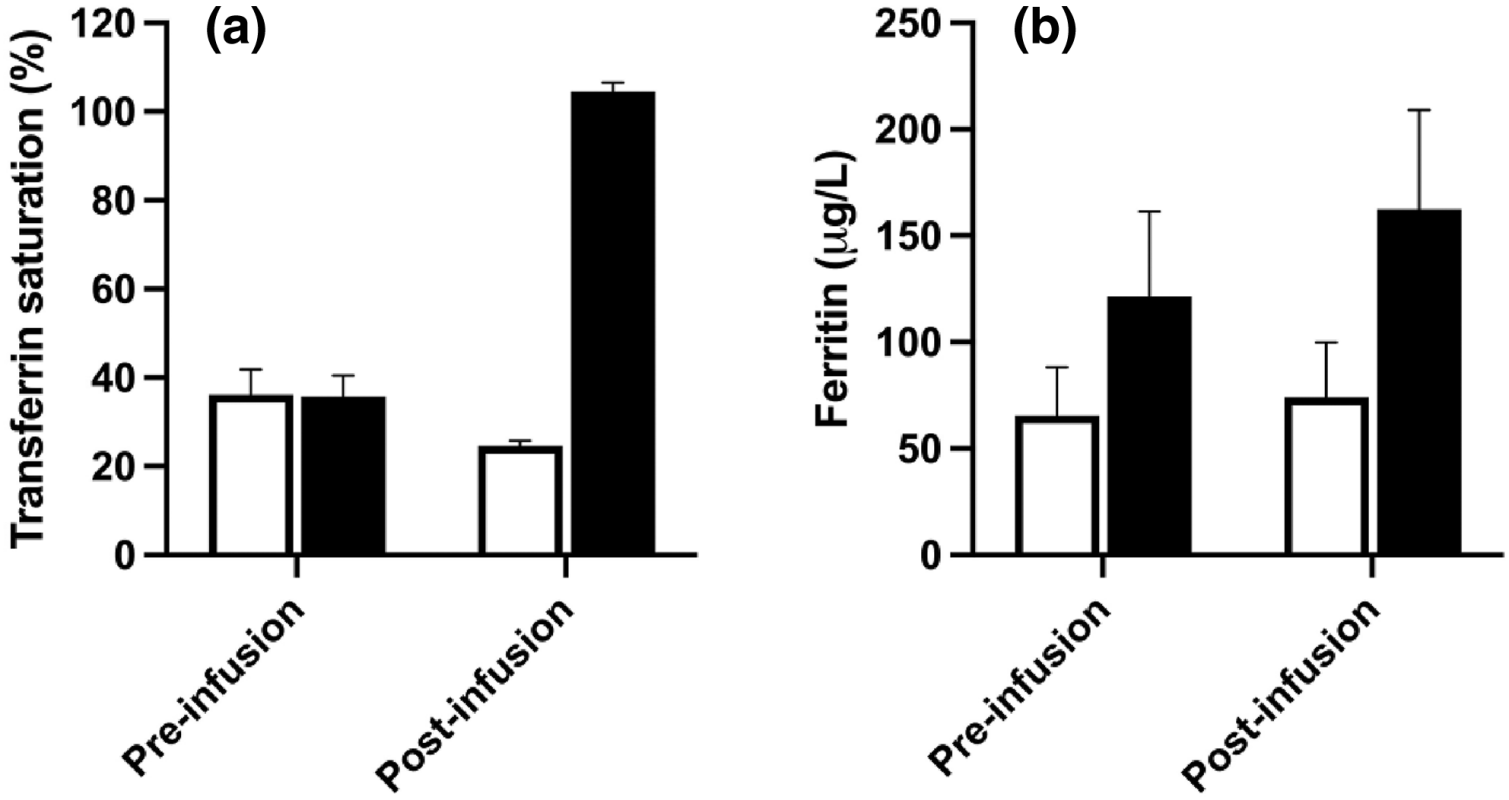
Serum Iron indices measured before and after infusion of iron sucrose (200 mg, black bars) or saline placebo (100 mL 0.9% saline, open bars). (a) Transferrin saturation, calculated from serum iron and transferrin saturation. (b) Ferritin. Bars indicate mean ± SEM. Note that apparent “oversaturation” of transferrin (transferrin saturation >100%) is known to occur following iron sucrose administration, due to the transient excess of serum iron.

Iron and saline infusions administered at the start of visit 3 were well tolerated. In the saline group, there was an apparent small fall in transferrin saturation by the end of visit 3, compared with the pre‐infusion values, with no change in ferritin concentration (Figure 4). The former is in keeping with known diurnal variation in serum iron, and did not reach statistical significance. In contrast, infusion of iron sucrose at visit 3 produced a substantial rise in both transferrin saturation (*p* < 0.001) and ferritin concentration (*p* < 0.01) by the end of the day (Figure 4).

**FIGURE 5:**
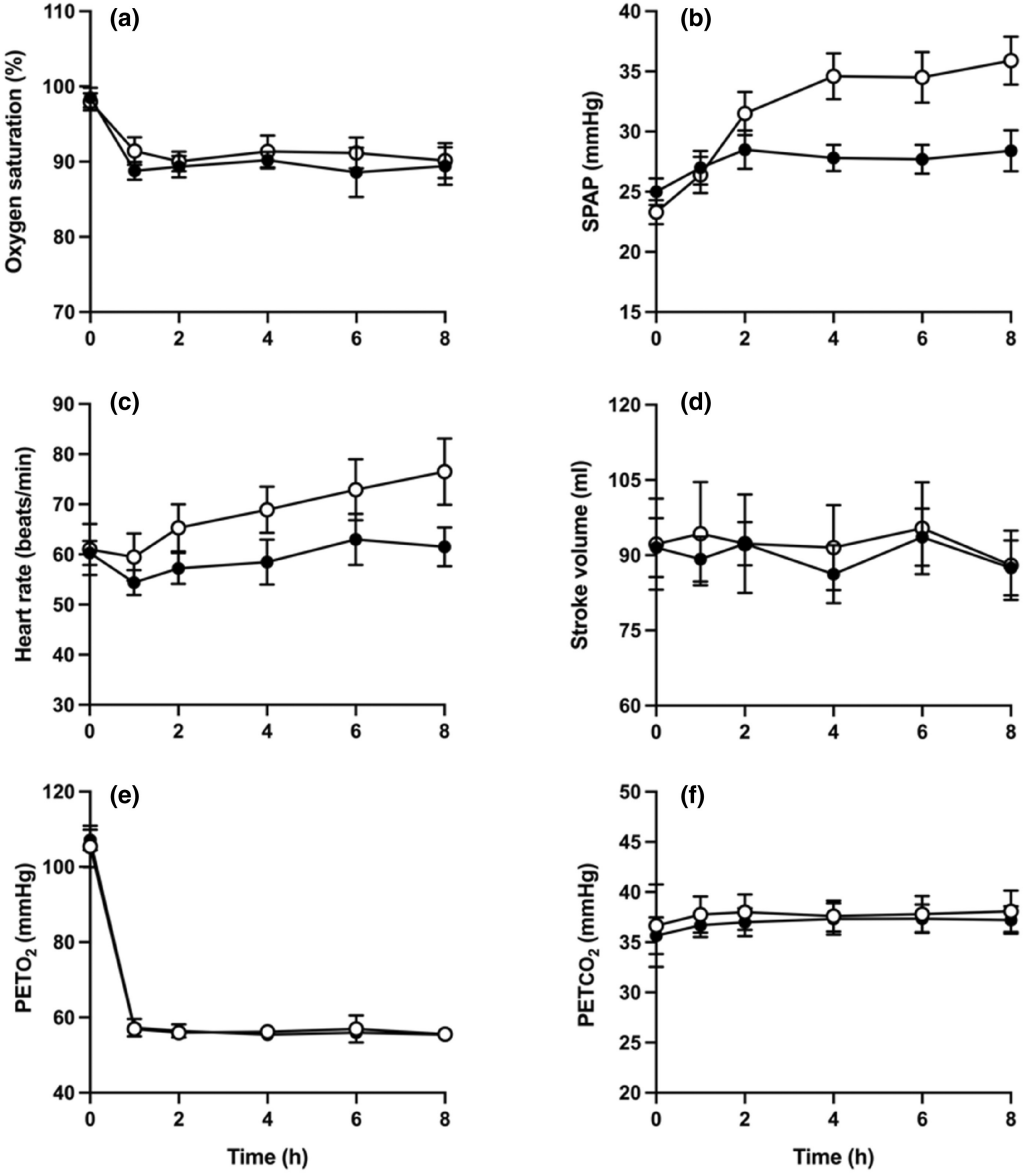
Exposure to 8 h of isocapnic hypoxia chamber (end‐tidal partial pressure of oxygen (PETO_2_) 55 mmHg, end tidal partial pressure of carbon dioxide (PETCO_2_) at each participant's normal value). (a) Peripheral oxyhemoglobin saturation. (b–d) Systolic pulmonary artery pressure, heart rate and stroke volume, respectively, estimated by echocardiography. (e, f) End tidal partial pressure of oxygen (PETO_2_) and carbon dioxide (PETCO_2_). Symbols represent mean ± SEM, with filled symbols representing those in the iron group and open symptoms representing those in the placebo group.

Figure 5 shows that the end‐tidal gas profiles and peripheral oxyhemoglobin saturation were very similar for the two groups during exposure to sustained isocapnic hypoxia. In the saline group, this was associated with a progressive rise in SPAP over the 8‐h exposure, with a mean increase of 12.6 ± 1.5 mmHg by the end of the exposure. In the iron group, the rise in SPAP over the first 2 h of the exposure was similar, but thereafter there was little further rise in SPAP, with a mean increase by the end of the exposure to hypoxia of just 3.4 ± 1.0 mmHg. This represents very substantial inhibition of the pulmonary vascular response to hypoxia by prior iron infusion, compared with saline (*p* < 0.01).

Figure 5 also shows changes in heart rate and stroke volume in the two groups during the chamber exposure. In the placebo group, heart rate rose by an average of 15.5 ± 5.2 beats/min, with a corresponding rise of 1.3 ± 4.6 beats/min in the iron group. However, the effect of iron on heart rate did not reach statistical significance (*p* = 0.177). Similarly, there was no effect of iron infusion on stroke volume (*p* = 0.610) or overall cardiac output (*p* = 0.377).

### 3.4 Effect of sustained hypoxia with and without iron on exercise capacity breathing 12% oxygen

**FIGURE 6:**
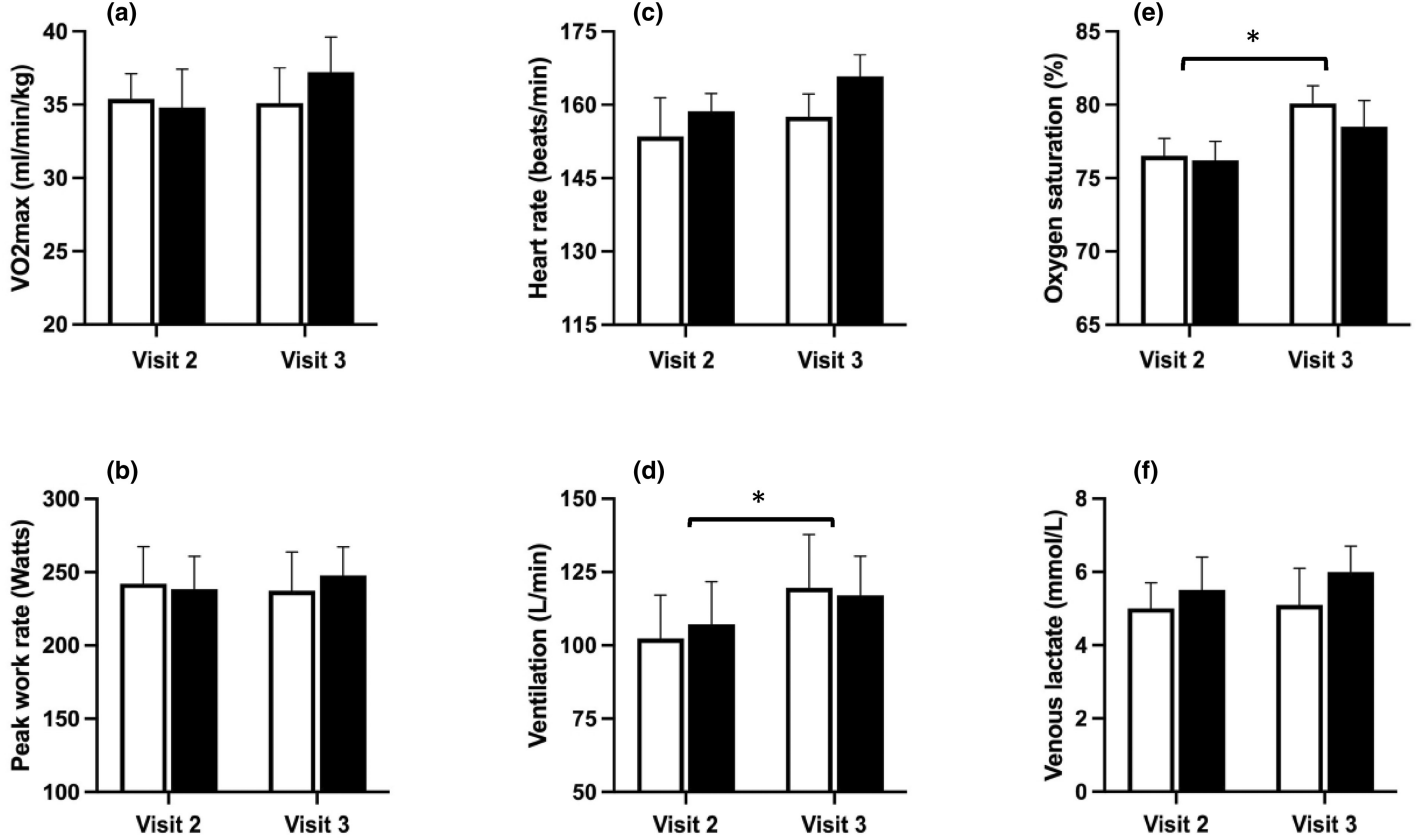
Exercise parameters before (visit 2) and after (visit 3) iron or saline infusion and 8 h of sustained isocapnic hypoxia. Open bars, saline group; filled bars, iron group. Parameters were measured during exercise testing breathing 12% oxygen. Maximal oxygen uptake (*V*O_2_max, a) and peak work rate (b) were determined using established methodology (Kuipers et al., 1985; Rossiter et al., 2006). Heart rate (c), ventilation (d), oxyhemoglobin saturation (e) and venous lactate (f) measurements were recorded at *V*O_2_max. In one participant at visit 2 and one participant at visit 3, saturation could not be accurately recorded for technical reasons. In two participants at visit 3, heart rate could not be accurately recorded for technical reasons. Bars show mean ± SEM. *Indicates a significant effect of visit on ventilation (*p* < 0.05) and oxyhemoglobin saturation (*p* < 0.01), based on repeated measures ANOVA.

As shown in Figure 6, despite the substantial rise in SPAP in the saline group during sustained hypoxia, there was no clear difference in *V*O_2_max breathing 12% oxygen during visit 3 (35.1 ± 2.4 mL/min/kg), compared with visit 2 (35.4 ± 1.7 mL/min/kg). In contrast, in the iron group *V*O_2_max was slightly higher during visit 3 (37.2 ± 2.4 mL/min/kg), compared with visit 2 (34.8 ± 1.7 mL/min/kg; *p* < 0.001). However, rmANOVA revealed no evidence of an overall difference between visits 2 and 3 (*p* = 0.285), and no evidence of an effect of iron infusion, compared with saline (*p* = 0.171 for interaction between visit and group).

There was also no evidence of an effect of sustained hypoxia on maximal work rate, heart rate and peak plasma lactate concentration on rmANOVA (*p* > 0.2 in each case), and no evidence of an effect of iron infusion on these parameters, compared with saline (*p* > 0.1 in each case, for interaction between visit and group).

Across both groups, there was evidence of a modest increase in ventilation during exercise at visit 3, compared with visit 2 (*p* < 0.05 for visit on rmANOVA), and of an associated increase in oxyhemoglobin saturation (*p* < 0.01), but there was no interaction between visit and group, suggesting no significant effect of iron infusion on these changes (*p* > 0.2 in both cases).

## 4 DISCUSSION

There were two main findings of this study. First, a period of sustained hypoxia produced a substantial elevation of pulmonary artery pressure in a group of healthy volunteers, but did not reduce maximal exercise capacity breathing 12% oxygen, compared with acute hypoxia alone. Second, intravenous iron infusion markedly inhibited the rise in pulmonary artery pressure during sustained hypoxia, but had only a modest effect on maximal exercise capacity during hypoxia, which was not significantly different from saline infusion.

It is well established that maximal exercise capacity falls when the inspired oxygen partial pressure is reduced, but the mechanism underlying this observation remains unclear. Much of the work in this area has been performed at high altitude, and it has been established in this setting that left ventricular function is preserved but left atrial pressure is reduced, compared with sea level exercise (Groves et al., 1987; Stembridge et al., 2015). This has led to the suggestion that right ventricular function may be a limiting factor at altitude, secondary to elevated pulmonary artery pressure (Naeije, 2011; Naeije & Chesler, 2012). In support of this possibility, pulmonary vasodilators including phosphodiesterase (PDE) inhibitors and endothelin antagonists have been shown to restore some of the fall in *V*O_2_max seen with acute hypoxia and at altitude (Faoro et al., 2009; Ghofrani et al., 2004; Naeije et al., 2010; Richalet et al., 2005). However, it is notable that not all studies have shown a beneficial effect of pulmonary vasodilatation on *V*O_2_max (Kressler et al., 2011; Toro‐Salinas et al., 2016), and in those that do, the effect size is modest (~30% improvement during acute hypoxia). In addition, in participants acclimatized to altitude, breathing 100% oxygen can restore *V*O_2_max close to baseline values, without normalizing pulmonary artery pressure (Anholm & Foster, 2011; Pugh et al., 1964), suggesting an important contribution from factors other than pulmonary hypertension.

In the current study, we used a novel approach to address the question of whether pulmonary artery pressure limits exercise during hypoxia. Specifically, rather than attempting to enhance *V*O_2_max through pulmonary vasodilatation and reducing right ventricular afterload, we sought to increase the pulmonary vascular sensitivity to hypoxia, by exposing volunteers to a period of sustained (8 h) isocapnic hypoxia at sea level. This approach has a number of advantages. First, it is known to produce a rapid and robust upregulation of pulmonary vascular sensitivity to hypoxia (Dorrington et al., 1997; Smith et al., 2008; Talbot et al., 2005, 2008). Second, it avoids the complicating factors associated with a prolonged period of exposure to high altitude, which might have multiple competing effects on exercise capacity. Finally, we have an established antagonist of this stimulus, in the form of intravenous iron (Smith et al., 2008; Talbot et al., 2014).

We hypothesized that the increased pulmonary vascular sensitivity induced by sustained hypoxia would be associated with greater right ventricular afterload during hypoxic exercise, and in turn with impaired left atrial filling and a reduction in maximal exercise capacity. However, in opposition to this hypothesis, we found no evidence that the magnitude of the pulmonary vascular response to hypoxia at rest predicts exercise capacity breathing 12% oxygen. We also observed a substantial fall in *V*O_2_max when breathing 12% oxygen acutely, despite there being very little time for the development of elevated pulmonary artery pressure. Finally, and most strikingly, we saw no reduction in *V*O_2_max or maximal work rate following sustained hypoxia, despite a substantial elevation of pulmonary vascular sensitivity.

Although there was a short (<20 min) period of normoxia between the end of the exposure to sustained hypoxia in the chamber and the start of hypoxic exercise, previous studies have shown that after 8 h of isocapnic hypoxia, the pulmonary artery pressure remains elevated for at least 2 h, and abrupt re‐exposure to hypoxia within this period leads to an exaggerated acute pulmonary vascular response (Dorrington et al., 1997). At the onset of hypoxic exercise, participants were all exposed abruptly to significant alveolar hypoxia, and as exercise progressed, they would also have experienced progressive mixed venous desaturation. We were not able to make measurements of SPAP during hypoxic exercise, but based on these considerations, we would expect the combination of alveolar and mixed venous hypoxia to provide a potent stimulus for hypoxic pulmonary vasoconstriction (Marshall et al., 1994).

Overall, we conclude that in this laboratory study, there was no evidence that elevated pulmonary artery pressure led to a reduction in maximal hypoxic exercise capacity. This conclusion is at odds with some of the studies cited above, but in keeping with a number of previous studies (Anholm & Foster, 2011; Toro‐Salinas et al., 2016). It also accords with the recent finding that although echocardiographic studies have suggested reduced right ventricular function at high altitude (Holdsworth et al., 2020; Stembridge et al., 2014, 2015), right heart catheterization and pressure‐volume analysis has recently shown preserved right ventricular function during acute exposure to 12% hypoxia in healthy volunteers, despite a significant increase in pulmonary artery pressure (Forbes et al., 2023). The reasons for the discrepancy between our findings and those from previous vasodilator studies are unclear, but they could relate to the nonspecific nature of PDE inhibitors and endothelin antagonists, which are reported to have effects on *V*O_2_max that are independent of right ventricular pressure, for example through increasing oxygenation (Rubin & Naeije, 2004). In this context, one possible factor contributing to the lack of any obvious detrimental effect of sustained hypoxia on *V*O_2_max in the current study is the increase in ventilation and arterial oxyhemoglobin saturation seen during hypoxic exercise after sustained hypoxia, presumably reflecting an increased ventilatory sensitivity to hypoxia.

In relation to iron, it is now well established that increased iron availability has an inhibitory effect on the pulmonary vascular response to hypoxia (Patrician et al., 2022; Smith et al., 2008, 2009; Willie et al., 2021). As in previous laboratory studies, the rise in SPAP over the first hour of exposure to sustained hypoxia in the current study was similar after iron and saline infusions (Smith et al., 2008; Talbot et al., 2014), but thereafter iron prevented almost entirely the gradual rise in SPAP associated with more prolonged exposure to hypoxia. This lag in the effect of iron would be in keeping with a proposed inhibitory effect of iron on the HIF transcriptional pathway, which coordinates systemic and cellular responses to hypoxia, including the pulmonary vascular and ventilatory responses (Bishop & Ratcliffe, 2015; Formenti et al., 2010; Hodson et al., 2016; Lakhal‐Littleton et al., 2019; Slingo et al., 2014; Smith et al., 2006). HIF levels are regulated by the prolyl hydroxylase domain (PHD) family of oxygen sensing enzymes (Ivan et al., 2001; Jaakkola et al., 2001), which are inhibited by low oxygen levels but are also regulated by iron availability. In the presence of hypoxia or restricted iron availability, PHD activity if inhibited, leading to HIF accumulation and activation of gene transcription. One known HIF‐regulated gene product is the vasoactive peptide endothelin‐1 (ET‐1), which is known to be upregulated during sustained hypoxia, and is established as a potent pulmonary vasoconstrictor (Cargill et al., 1995; Goerre et al., 1995). It has recently been shown that ET‐1 levels are lower during sustained hypoxia following an iron infusion, compared with saline (Lakhal‐Littleton et al., 2019).

In the current study, despite a substantial inhibition of the pulmonary vascular response to hypoxia following iron infusion, our primary analysis found no evidence that inhibiting the pulmonary vascular response to hypoxia by iron had a beneficial effect on exercise capacity during hypoxia. This accords with the lack of an effect of sustained hypoxia on *V*O_2_max in the saline group, and with the conclusion that pulmonary artery pressure is unlikely to be limiting *V*O_2_max in acute hypoxia, at least in healthy volunteers. However, although there was no statistical difference between iron and saline infusion, it is of note that there was a small rise in *V*O_2_max at visit 3 in the iron group, compared with visit 2. For the reasons discussed above, this seems unlikely to be related directly to the effects of iron on SPAP. Other possible explanations include improvements in gas exchange, as has been suggested for other pulmonary vasodilators (Faoro et al., 2009; Rubin & Naeije, 2004), or indeed for iron at very high altitude (Holdsworth et al., 2020), but the rise in oxyhemoglobin saturation was if anything slightly lower in the iron group, compared with saline. It has also been suggested in previous studies that iron may enhance exercise capacity through effects on skeletal muscle metabolism. In a group of healthy iron‐replete volunteers undertaking submaximal exercise at sea level, for example, prior iron infusion increased lactate threshold (Frise et al., 2022). Metabolic effects of iron may also be mediated through the HIF‐pathway, which is known to regulate the balance between oxidative and glycolytic metabolism (Aragones et al., 2008; Formenti et al., 2010; Mason et al., 2004). Given the long‐lasting effects of intravenous supplementation on systemic iron availability, it is possible that the full extent of any such metabolic adaptation would not have been evident within the relatively short time frame of the current study.

This study has a number of other limitations. From a technical perspective, there was a small amount of missing data, due primary to technical difficulties relating to recording of oxyhemoglobin saturation and heart rate during exercise in a small number of individuals. This limits the confidence with which differences in these parameters between groups can be interpreted, but has no impact on the primary outcome/conclusions of the study. In addition, meaningful measurements of SPAP during maximal exercise were not possible during heavy exercise, due to the inherent technical limitations of this technique. From a design perspective, our statistical power is limited by small numbers in the two groups (iron and saline), which in turn reflects the unpaired design of the study. This was felt to be necessary for two primary reasons. First, the effect of iron in the pulmonary circulation is very long‐lasting, with significant inhibition of the pulmonary vascular response to hypoxia evident for at least 43 days following a single infusion of iron ferric carboxymaltose (Bart et al., 2016). This effectively precludes a cross‐over design. Second, there may be learning effects in participants not accustomed to maximal exercise testing (Astrand, 1976), which would complicate a paired design in which the saline infusion was always given prior to the iron infusion. A further limitation is the small number of female participants, which reduced our ability to identify any differences between male and female participants. No such differences were evident in the current study, but this should be explored in future studies, particularly given the potential impact of menstruation and pregnancy on iron status. Finally, a strength of our study was the relatively homogenous nature of our participant group, and the well‐controlled laboratory setting. However, this limits our conclusions to healthy young volunteers exposed to acute hypoxia, and complicates direct comparison with results obtained after more prolonged exposure to hypoxia at altitude. In addition, our conclusions may not apply to older participants, in whom both hypoxia and exercise may be associated with larger rises in pulmonary artery pressure (Balanos et al., 2015; Taylor & Johnson, 2010), or in patients with cardiorespiratory disease, in whom iron deficiency is common (Nickol et al., 2015; Ruiter et al., 2011; van Veldhuisen et al., 2011). Indeed, intravenous iron has been shown to reduce pulmonary artery pressure during submaximal exercise in healthy older participants (Cheng et al., 2019), and to enhance submaximal exercise capacity in patients with COPD or heart failure (Anker et al., 2009; Santer et al., 2020). Further research on the effects of iron on exercise performance in these groups is warranted.

## AUTHOR CONTRIBUTIONS

N.P.T., T.G.S., K.L.D. and P.A.R. conceived and designed the experiments. H‐Y. C., H.H., T.G.S., K.L.D. and N.P.T. were involved in data acquisition. N.P.T. analyzed the data and drafted the manuscript. All authors reviewed and approved the final version of the manuscript, and agree to be accountable for all aspects of the work. All persons designated as authors qualify for authorship, and all those who qualify for authorship are listed. The experiments were conducted in the Department of Physiology, Anatomy and Genetics at the University of Oxford, United Kingdom.

## FUNDING INFORMATION

The study was funded by the Oxfordshire Health Services Research Committee (OHSRC, 968). H‐Y.C. was the recipient of a Swire Scholarship. N.P.T. was the recipient of a National Institute of Health and Care Research (NIHR) Academic Clinical Fellowship. The views expressed are those of the author(s) and not necessarily those of the NIHR or the Department of Health and Social Care.

## CONFLICT OF INTEREST STATEMENT

P.A.R. has received funding for basic science on iron homeostasis from Vifor Pharma. The other authors have no competing interests to declare.

## Data Availability

The data that support the findings of this study are available from the authors on reasonable request.
